# Navigating ethical minefields: a multi-stakeholder approach to assessing interconnected risks in generative AI using grey DEMATEL

**DOI:** 10.3389/frai.2025.1611024

**Published:** 2025-07-01

**Authors:** Sridhar Jonnala, Nisha Mary Thomas, Sarthak Mishra

**Affiliations:** ^1^IBM India Pvt. Ltd., Bengaluru, India; ^2^International School of Management Excellence, Bengaluru, India; ^3^Symbiosis Institute of Business Management, Bengaluru, India

**Keywords:** generative AI, foundation models, ethical AI, AI risks, responsible AI

## Abstract

The rapid advancement of generative artificial intelligence (AI) technologies has introduced unprecedented capabilities in content creation and human-AI interaction, while simultaneously raising significant ethical concerns. This study examined the complex landscape of ethical risks associated with generative AI (GAI) through a novel multi-stakeholder empirical analysis using the grey decision-making-trial-and-evaluation-laboratory methodology to quantitatively analyze the causal relationships between risks and their relative influence on AI deployment outcomes. Through a comprehensive literature review and expert validation across three key stakeholder groups (AI developers, end users, and policymakers), we identified and analyzed 14 critical ethical challenges across the input, training, and output modules, including both traditional and emerging risks, such as deepfakes, intellectual property rights, data transparency, and algorithmic bias. This study analyzed the perspectives of key stakeholders to understand how ethical risks are perceived, prioritized, and interconnected in practice. Using Euclidean-distance analysis, we identified significant divergences in risk perception among stakeholders, particularly in areas of adversarial prompts, data bias, and output bias. Our findings contribute to the development of a balanced ethical risk framework by categorizing risks into four distinct zones: critical enablers, mild enablers, independent enablers, and critical dependents. This categorization promotes technological advancement and responsible AI deployment. This study addressed the current gaps in academic work by providing actionable recommendations for risk-mitigation strategies and policy development while highlighting the need for collaborative approaches among stakeholders in the rapidly evolving field of GAI.

## 1 Introduction

Artificial intelligence (AI) has evolved significantly since its inception, transitioning from simple rule-based systems to complex machine-learning models capable of performing sophisticated tasks. Early AI systems, such as the expert systems of the 1970s and the 1980s, relied on predefined rules to mimic human decision-making. The advent of machine learning in the 1990s marked a paradigm shift, enabling systems to learn patterns from data without explicit programming (Russell and Norvig, 2020). This evolution provided the foundation for deep-learning research in the 2010s, leading to the development of neural networks capable of tackling complex problems, such as image recognition, speech processing, and natural language understanding.

Generative AI (GAI) represents a significant leap in AI capabilities, focusing on creating new and contextually relevant outputs, rather than simply analyzing or classifying data. Unlike traditional AI, which operates within predefined parameters, GAI leverages models such as generative adversarial networks (GANs), variational autoencoders (VAEs), and transformer-based architectures [e.g., generative pre-trained transformer (GPT) and (DALL-E)]. These models are designed to generate original content by learning the underlying structure and patterns in training datasets (Goodfellow et al., 2016).

- Text generation: Large Language Models (LLMs) like GPT-4 excel at generating coherent and contextually relevant text for applications such as chatbots, content creation, and summarization.
- Image generation: Models like DALL-E and Stable Diffusion create photorealistic images from textual descriptions, enabling creative applications in design and marketing.
- Video and audio generation: Tools like Synthesia and WaveNet produce realistic videos and speech, revolutionizing multimedia production.
- Code generation: Models like Codex generate programming code, enhancing developer productivity and enabling non-programmers to create software solutions.
- Music and art creation: Generative AI tools such as MuseNet and DeepArt produce original compositions and artworks, democratizing creativity.

The versatility of GAI has led to its integration into diverse domains.

- Healthcare: According to Lee (2023), generative AI is used in healthcare for synthesizing medical images, generating personalized advice, and aiding drug discovery.
- Education: Automated content creation for personalized learning experiences. Entertainment: Developing game content, creating immersive virtual environments, and generating scripts.
- Business: Enhances customer service through chatbots, automates report generation, and optimizes marketing strategies.

However, despite its transformative potential, the rapid deployment of GAI has raised significant ethical and security concerns. GAI introduces a range of risks that span technical, ethical, and social domains. Technical risks include adversarial prompts, in which malicious inputs manipulate model behavior (Wang et al., 2023), and hallucinations, in which models generate plausible but factually incorrect outputs (Huang et al., 2025). Vulnerabilities in the software and hardware, such as prompt injection attacks (Rossi et al., 2024) and side-channel exploitation (Jalil, 2024), add to the complexity. Ethical risks include bias and discrimination from unbalanced training data (Weidinger et al., 2021), the generation of inappropriate or harmful content owing to not-safe for-work (NSFW) material (Europol, 2023), and copyright infringement concerns from replicating protected content (Carlini et al., 2023). Societal risks involve the creation of misinformation and phishing schemes that pose threats to public trust and security, while privacy concerns emerge from inadvertent access to sensitive information (Feretzakis et al., 2024). These interconnected risks highlight the need for a holistic approach to understanding and mitigating their implications for responsible deployment.

Although substantial research has been conducted on the risks posed by AI and GAI, the focus of existing studies has generally been on individual risks in isolation (Wang et al., 2023). There is a critical gap in the understanding of the interrelationships and causal effects between different risks. For example, data bias can exacerbate output bias, leading to discriminatory results, whereas hallucinations may compound the issue of misinformation when unchecked. Addressing these interconnected risks requires a holistic approach for examining how they interact within a broader AI ecosystem.

This study addresses these gaps by pursuing three primary research questions.

1. How do stakeholders (developers, end users, and policymakers) perceive and prioritize ethical risks in AI?
2. What are the causal relationships between different risk enablers, and how do they influence system-wide vulnerabilities?
3. How can understanding these relationships inform the development of effective risk mitigation strategies?

Market context underscores the urgency of this study. Recent industry reports indicate that the adoption of GAI has grown exponentially, reaching USD 196.63 billion in 2023 and is projected to grow at a compound annual growth rate (CAGR) of 36.6 from 2024 to 2030 (Grand View Research, 2024). Continuous research and innovation directed by technology giants are driving the adoption of advanced technologies in industry verticals, such as automotive, healthcare, retail, finance, and manufacturing. This rapid adoption amplifies the need for a structured approach to understanding and managing the associated risks.

Our study employs the grey Decision-Making Trial and Evaluation Laboratory (DEMATEL) methodology (Rajesh and Ravi, 2017), which offers distinct advantages over traditional analytical approaches. The ability of this method to handle uncertainty in expert opinions and quantify complex causal relationships makes it particularly suitable for analyzing interconnected risks in emerging technologies (Thomas, 2023). The multi-stakeholder approach provides a comprehensive view of risk perception and prioritization, addressing a critical limitation of existing studies that often focus on single-stakeholder perspectives.

The significance of this study lies in its practical implications for AI governance and development. By analyzing both traditional and emerging risks through the lens of different stakeholders, this study contributes to the following:

1. Development of more effective risk-mitigation strategies aligned with stakeholder concerns.
2. Creation of balanced regulatory frameworks that promote innovation while ensuring ethical compliance.
3. Enhancement of trust in GAI systems through improved risk management.

This study emphasizes the interconnected nature of ethical risks and their varying impacts across the different modules of GAI systems. Understanding these relationships is crucial for developing comprehensive risk-mitigation strategies that address the direct and indirect effects of ethical challenges.

## 2 Taxonomy of risks

The emergence of LLMs has revolutionized natural language processing (NLP) capabilities, enabling unprecedented advances in text generation, summarization, and conversational AI. These models have been rapidly integrated into critical domains such as healthcare, education, and finance, transforming workflows and decision-making processes. However, this transformative potential is coupled with significant security, ethical, and societal concerns that require urgent and careful consideration. Understanding and addressing these inherent risks are essential for ensuring the secure and responsible deployment of LLMs in real-world applications (Weidinger et al., 2021).

Previous research has established various frameworks for categorizing AI risks – from operational risks (Amodei et al., 2016) to ethical concerns (Floridi, 2020). However, GAI introduces unique challenges that require an expanded framework. This study builds upon traditional risk categorization approaches, while incorporating novel dimensions specific to generative systems.

Among the most pressing risks are adversarial prompts, in which malicious actors exploit inputs to manipulate LLM behavior and exploit vulnerabilities. These attacks bypass safety mechanisms and trigger unwanted or harmful outputs, such as misinformation or content-moderation filters (Wang et al., 2023; Nasr et al., 2024). Addressing this issue requires advanced adversarial training techniques and robust input-validation methods to enhance model resilience. Similarly, the challenge of managing NSFW risks persist owing to the inclusion of inappropriate or offensive material in the training datasets. The outputs generated from such content can be harmful, particularly for professional or public-facing applications (Weidinger et al., 2021). Stricter dataset curation and postprocessing techniques are crucial for mitigating these risks and ensuring that the outputs are in line with ethical standards.

Improper handling of confidential data in prompts introduces a critical privacy risk. LLMs can inadvertently memorize and expose sensitive information, leading to data leakages and privacy violations during inferences or interactions (Feretzakis et al., 2024). This risk is compounded by membership inference attacks and training-data extraction, which allow adversaries to retrieve sensitive details from models (Nasr et al., 2024; Carlini et al., 2023). Implementing privacy preserving techniques such as differential privacy and encryption is essential for protecting user and organizational data.

Data and output biases are interconnected challenges that stem from imbalanced training datasets which reflect societal inequities. These biases manifest as discriminatory responses or reinforce stereotypes based on gender, race, and socioeconomic status (Weidinger et al., 2021). Addressing this bias requires the inclusion of diverse datasets and continuous monitoring during training and evaluation to ensure fairness and equity.

The issue of data-usage rights further complicates this landscape. LLMs often rely on publicly scraped datasets, which raises questions regarding intellectual property, consent, and ethical use (Carlini et al., 2023). Ensuring compliance with data-usage laws and ethical guidelines is critical for addressing these challenges and preventing legal disputes.

The black-box nature of LLMs poses another challenge in building accountability and trust. The lack of transparency in the model hinders the understanding of decision-making processes, particularly in high-stakes domains such as healthcare or finance (Remmer, 2022). Enhancing.

transparency through techniques such as attention-based mechanisms, retrieval-augmented generation, and layer-wise relevance propagation can improve interpretability and foster trust among users (Nikiforidis et al., 2024).

From a technical perspective, software-security issues such as prompt-injection attacks, insecure code generation, and exploitative scripts present significant risks. For example, malicious actors can manipulate LLMs to bypass security protocols or generate harmful codes (Rossi et al., 2024; Jalil, 2024). Hardware vulnerabilities such as side-channel attacks, create new attack surfaces during model training and inference (Jalil, 2024). Robust security protocols, including hardware encryption and secure inference environments, are essential to mitigate these risks.

Copyright infringement is another critical concern because LLMs can reproduce or closely mimic copyrighted content without appropriate attribution (Weidinger et al., 2021), raising questions about the intellectual property and fair use of AI-generated content, thus necessitating clear policies and technical safeguards, such as content watermarking.

The phenomenon of hallucination poses serious challenges in applications requiring precision and reliability, such as legal and medical advice. Hallucinations occur when models generate plausible but incorrect information that can have serious consequences (Huang et al., 2025). Retrieval-augmented-generation approaches, and factual-consistency mechanisms have shown promise in reducing hallucinations.

Toxic outputs, in which LLMs generate offensive or harmful content, remain a persistent issue despite advancements in content moderation. These outputs can fuel misinformation and social-engineering attacks, highlighting the need for robust safeguards (Europol, 2023). Furthermore, the improper use of LLMs, such as generating phishing emails, fake news, or malicious scripts, presents significant security risks that require strong ethical guidelines and access controls (Recorded Future, 2024).

Finally, the challenge of explaining outputs affects trust and accountability in LLM applications. Techniques such as rationale generation, layer-wise inspections, and decision-path tracking are essential for improving the model accuracy (Nikiforidis et al., 2024). Hagendorff (2024) also emphasizes the importance of ethical transparency in model development. These advances are critical for fostering user confidence and ensuring the responsible adoption of LLM technologies.

We analyzed previous studies and their limitations (Hagendorff, 2024; Weidinger et al., 2021) with a particular focus on identifying and categorizing AI risks. Existing research often treats risks as isolated issues and fails to capture their interconnected and cascading nature—critical for developing effective mitigation strategies. To address this gap, we developed a comprehensive taxonomy that systematically classifies GAI applications. This new GAI risk-taxonomy framework provides a robust method for understanding applications and associated risks and models the interdependencies and feedback loops among risks, offering a more holistic approach to addressing vulnerabilities across multiple dimensions of GAI systems.

**Figure 1:**
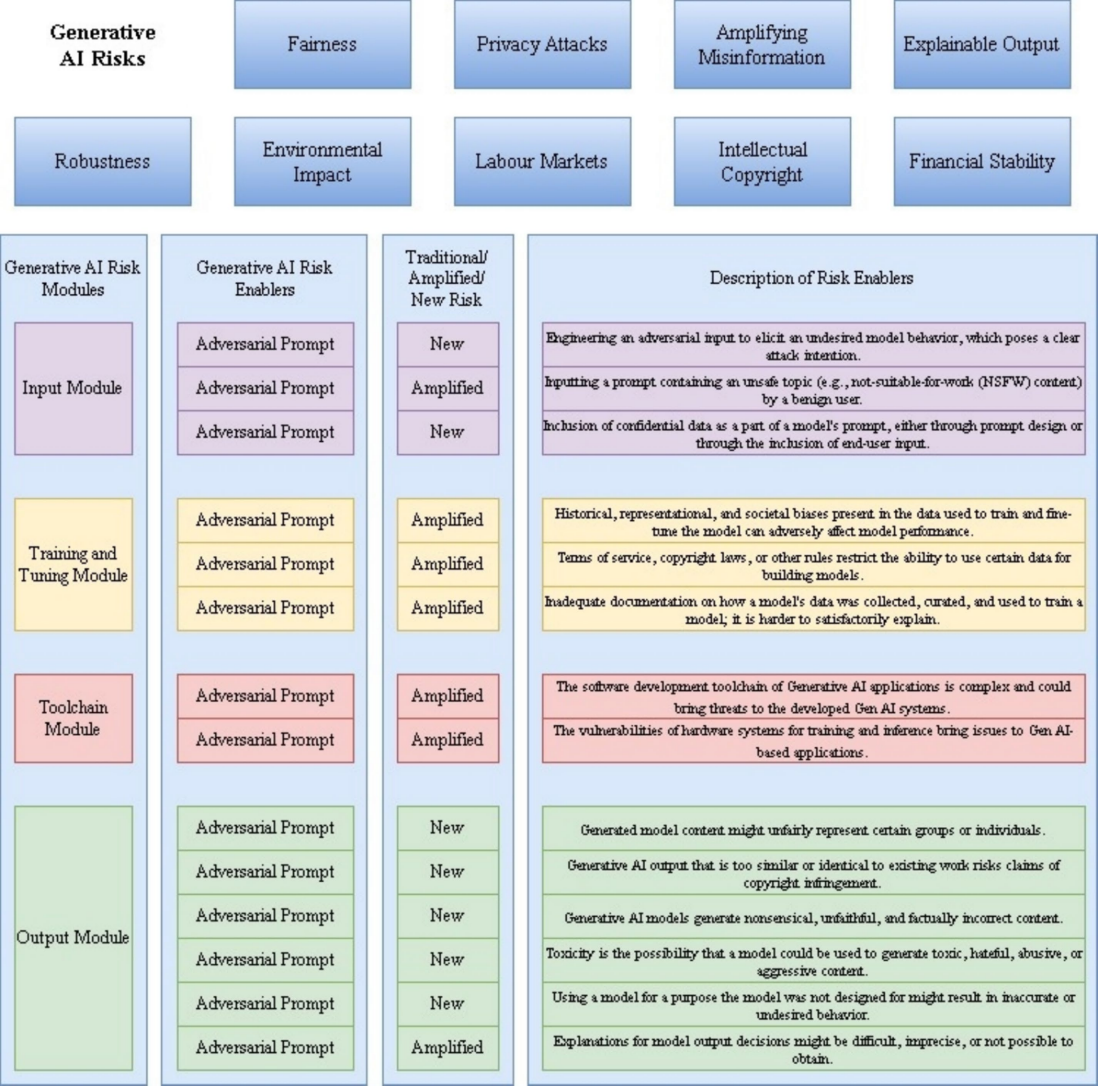
Taxonomy of generative AI risk enablers.

Figure 1 presents the overarching framework of our taxonomy for GAI risk, which consists of three key layers: (1) Core Modules: Input, Training and Tuning, Toolchain, and Output modules that represent the fundamental components where risks originate; (2) Risk Categories: Traditional/Amplified risks versus New risks specific to GAI; and (3) Risk Enablers: Factors that contribute to or amplify risks across modes, which enable systematic risk analysis while acknowledging the interconnected nature of GAI risks. Unlike traditional AI risk frameworks that focus primarily on output-based risks (Xiaoge et al., 2022), our taxonomy emphasizes the propagation of risks across different modules and their mutual influence.

The modular structure of the framework reflects the unique characteristics of GAI systems; the module-specific risks include

- Input module: Focuses on prompt-based risks and data-injection vulnerabilities.
- Training and tuning module: Addresses Model Architecture and Learning Process Risks.
- Toolchain module: Covers deployment and integration risks.
- Output module: Encompasses generation and usage risks.

Each module interfaces with the others through specific risk enablers, creating potential cascade effects. This interconnected structure builds on earlier work in AI risk modeling (Mitchell et al., 2019), while incorporating GAI-specific elements and explicitly mapping how risks propagate across modules. For example, the data bias in the Input Module can be amplified through the Training Module, ultimately manifesting as biased outputs. This propagation mapping extends previous work on AI risk chains; consequently, the developed taxonomy comprehensively addresses the risks associated with both traditional AI systems and GAI models. Table 1 presents the risk enablers and their detailed explanations.

**Table 1:** Total relation matrix for AI developer risk enablers.

Risk enablers	Denotation	Explanation
Adversarial prompts	RE1	Deliberately crafting prompts to trick the AI into generating harmful or misleading responses. For example, manipulating a text generator to produce offensive content by subtly tweaking the input prompts.
Not suitable for work prompts	RE2	Submitting prompts that lead to the generation of content inappropriate for professional or general settings, such as explicit adult content or graphic violence. Example: A user requests a generative AI to create an image or story with explicit themes.
Confidential data in prompts	RE3	Accidentally or intentionally using sensitive or confidential information in prompts, which risks exposing this data in the AI’s output. Example: Including personal identification numbers or proprietary business information in a data processing request.
Data bias	RE4	The presence of prejudiced or skewed data in training sets that lead the AI to generate biased outputs. For example, a language model trained on biased historical texts may produce stereotypical or discriminatory language.
Data usage rights	RE5	Using data in ways that violate copyrights, licensing agreements, or terms of service. Example: A company uses copyrighted images to train an AI without obtaining proper permissions.
Model transparency	RE6	The lack of clear, accessible information on the AI model’s workings can prevent users from fully understanding or trusting its decisions. For example, if a financial advisory AI provides investment suggestions without explaining the reasoning, users may be hesitant to follow the advice.
Software security issues	RE7	Vulnerabilities in the software tools used to develop, train, or deploy AI models can lead to security risks. An example is an AI development platform that is susceptible to code injections that could alter model behavior.
Hardware vulnerabilities	RE8	Physical or firmware vulnerabilities in the hardware used for AI operations could compromise the entire system. For instance, unauthorized access to a server’s hardware could lead to the theft or alteration of AI data.
Output bias	RE9	AI outputs that disproportionately favor or disfavor certain groups due to biases in the data or model. An example is a recruitment AI that favors candidates from a particular demographic due to biased training data.
Copyright infringement	RE10	AI-generated content that inadvertently copies or closely mimics copyrighted works without permission. For example, an AI that composes music may create a piece that closely resembles a copyrighted song.
Hallucination	RE11	AI models producing outputs that are completely unfounded or factually incorrect, often due to overfitting or data quality issues. An example is a historical AI generating an inaccurate event that never occurred.
Toxic output	RE12	AI models outputting content that could be considered offensive, discriminatory, or harmful. This might occur in response to certain inputs or because of biases in the training data, such as an AI generating hate speech.
Improper usage	RE13	Applying AI models in contexts they were not designed for, potentially leading to inaccurate or unsafe outcomes. For example, using a model trained on adult fiction to generate children’s stories.
Explaining output	RE14	Inadequate explanations for the decisions made by AI models can make it difficult for users to understand or validate the results. For instance, a medical diagnosis AI failing to provide reasons for its conclusions could lead to trust issues among healthcare professionals.

## 3 Methodology

### 3.1 Research framework

**Figure 2:**
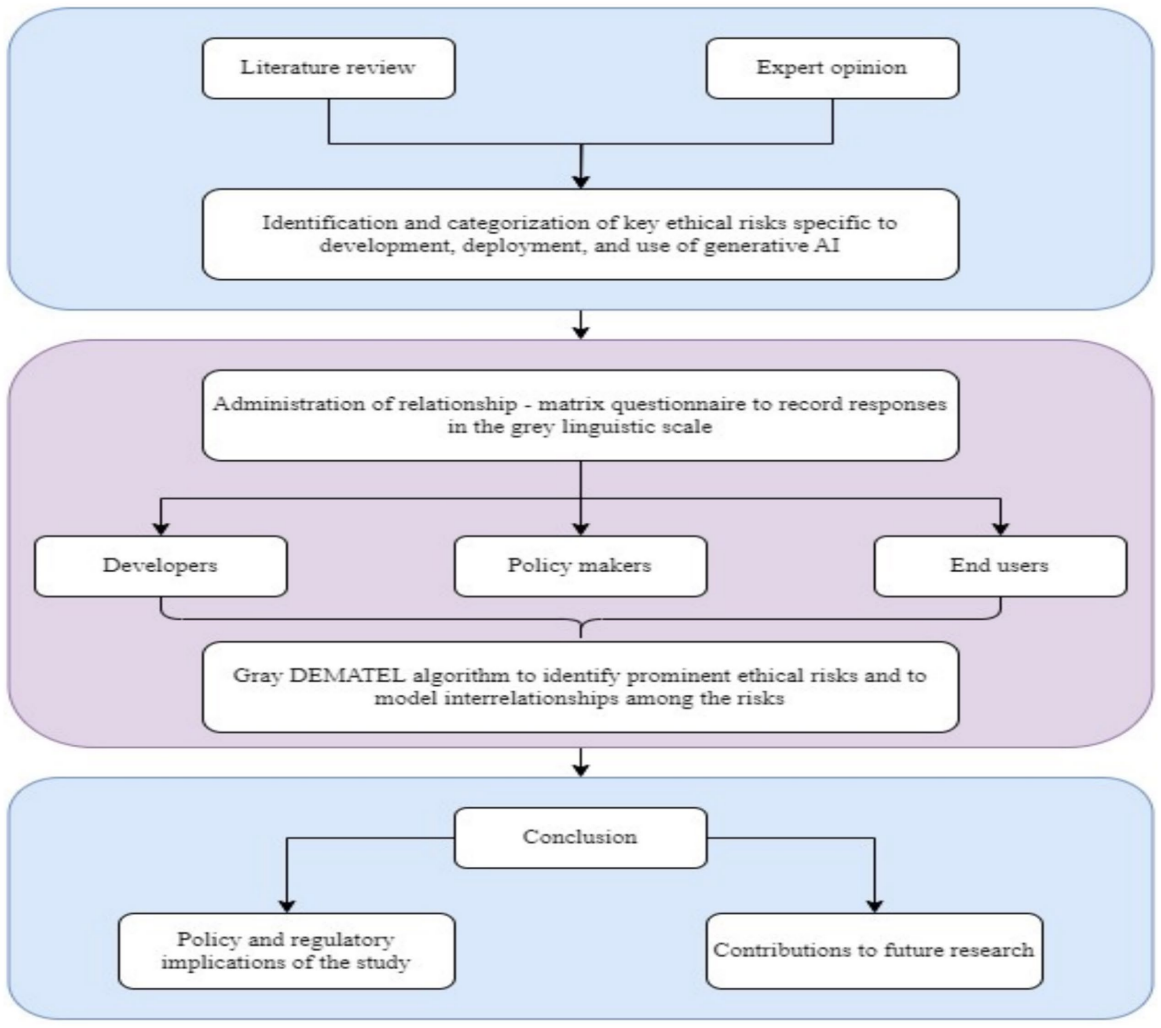
Research methodology.

This study employs the structured research framework shown in Figure 2 to systematically identify and evaluate the interrelationships among GAI risks. The framework is structured into three phases: (Carlini et al., 2023) literature review and risk identification, (Crawford et al., 2019) data collection and stakeholder perception analysis, and (Amodei et al., 2016) interrelationship modeling using the Grey DEMATEL method. This multiphase approach ensures a comprehensive understanding of GAI risks and their cascading effects across various modules.

### 3.2 Literature review and risk identification

The first phase involved an extensive literature review to identify key ethical and technical risks associated with GAI systems. These risks were categorized into three modules—Input, Training & Tuning, and Out output/usage – encompassing traditional AI risks and those specific to GAI. The identified risks include adversarial prompts, data bias, hallucinations, toxic outputs, and security vulnerabilities. To validate the relevance of these risks, feedback was solicited from industry experts, ensuring that the final taxonomy reflected both theoretical and practical perspectives—covered in detail in the Taxonomy of Risks section.

### 3.3 Data collection and stakeholder analysis

#### 3.3.1 Study design

A cross-sectional study design incorporating a multistakeholder approach was used. This multistakeholder approach captures diverse perspectives on the risks associated with GAI from key stakeholders, including AI developers, policymakers, and end users, thus providing a holistic understanding of risk prioritization and perceptions.

#### 3.3.2 Sample characteristics

The study sample (*N* = 45) was divided equally among the three stakeholder groups, with 15 participants in each group. The participants were purposively sampled to ensure diversity in geographical location, demographic background, professional expertise and availability. Direct invitations were extended to stakeholders meeting these criteria, ensuring alignment with the study’s objectives.

#### 3.3.3 Data collection instruments

A standardized questionnaire was designed, based on the finalized taxonomy of GAI risks. The questionnaire used a six-point linguistic scale ranging from “no influence” to “very high influence” to measure stakeholder perceptions of the relationships among risks, thus facilitating quantitative and qualitative analyses of the degree and direction of influence among the risks. The questionnaire was structured as a standardized Excel template to ensure clarity and consistency. This template included predefined risk enablers (RE1–RE14) with definitions, a matrix for pairwise influence evaluations, and practical examples—such as assessing how “toxic outputs” amplify “improper usage”—to guide participants in interpreting and scoring risks.

#### 3.3.4 Data collection procedure

To understand the risk impacts on different stakeholders, we analyzed three key respondent categories: AI developers, policymakers, and end users.

- AI developer: AI developers focus on creating robust, ethical, and secure AI models. The risks relevant to developers include adversarial prompts, data bias, and security. Developers need guidelines and safeguards to mitigate risks related to unintentional bias, security threats, and confidential data use.
- AI policy maker: AI policymakers ensure that AI systems align with regulatory standards. The key risks include transparency, data usage rights, and output bias. Policymakers should focus on regulatory compliance, data protection, and content moderation to foster accountability and protect intellectual property.
- AI end user: End users interact directly with the AI output without understanding the underlying mechanisms. These risks include toxic outputs, NSFW content, and lack of transparency. For example, biased hiring practices can arise from an output bias, underscoring the need for safeguards.

The selection of stakeholders, developers, AI policy, and end users aligns with the diverse responsibilities and impacts of GAI risks across the AI ecosystem. According to Crawford et al. (2019), understanding developer perspectives is essential because developers are responsible for embedding ethical considerations into AI design, particularly addressing risks such as data bias and adversarial prompts. Policymakers play a critical role in ensuring compliance with ethical standards and regulatory frameworks, as highlighted by Floridi (2020), who emphasizes the need for accountability, transparency, and data protection. End users, who are often directly affected by AI outputs, face risks such as toxic content and a lack of transparency, which can result in real-world consequences such as discriminatory hiring practices or misinformation, as noted by Mittelstadt et al. (2016). Including these stakeholders provides a holistic understanding of GAI risks and fosters solutions tailored to unique roles and challenges.

This study involved 45 participants from three key stakeholder groups to ensure a comprehensive understanding of GAI risks. These groups included 15 developers, comprising AI engineers, data scientists, and researchers from major AI organizations involved in the design and deployment of GAI models. Another 15 participants were end users, representing professionals across industries, such as healthcare, finance, and education, who used GAI tools in their work. In addition, 15 policymakers and regulators, including government advisors, representatives from global regulatory bodies, and legal experts specializing in AI ethics and governance, participated in this study. The Excel template with the questionnaire was shared with participants via email allowing them to complete it at their own pace. We provided initial guidance through briefings to clarify terminology and scoring methodology. Participants were encouraged to seek clarifications during the process, with iterative support provided via email and multiple follow-up discussions. Each participant independently assessed the influence of one risk over another using the provided scale. Ethical considerations including informed consent and participant confidentiality were strictly adhered to throughout the data collection process. This diverse composition ensures the inclusion of technical, practical, and regulatory perspectives, thereby providing a well-rounded analysis of the challenges and opportunities associated with GAI systems. By capturing insights from these stakeholder groups, this study aimed to develop actionable solutions to mitigate risks and foster ethical AI practices.

### 3.4 Modeling interrelationships: application of the grey DEMATEL method

The grey DEMATEL method was employed to model and analyze the interrelationships among the identified risks. This method was selected because of its ability to achieve effective risk management (requiring a comprehensive approach that addresses multiple interconnected challenges). First, it must account for the inherent ambiguity and uncertainty associated with human decision making and assessment. Second, establishing clear mathematical or quantitative frameworks is necessary to map and understand how different risks influence and relate to each other through causal relationships. Finally, a systematic method for risk standardization should be implemented that considers both the individual significance of each risk and how it connects to and influences other risks in the system. This integrated approach facilitates a robust and practical risk-management strategy that can adapt to complex real-world scenarios. The following steps were undertaken:

- Construction of initial relation matrices: Pairwise influence assessments were aggregated into an initial-relationship matrix for each stakeholder group.
- Computation of grey relation matrices: The linguistic assessments were converted into grey numbers to reflect the range of influence values.
- Crisp relation matrix: Grey numbers were converted into crisp values to standardize the relationship matrix.
- Total relation matrix: A total relation matrix was computed to represent the direct and indirect influences of risks.
- Prominence and causality analysis: The total relationship matrix was analyzed to identify prominent risks (those with the highest combined influence) and their causal or effect driven nature.
- Threshold setting: A threshold value was applied to filter significant causal relationships and reduce complexity while retaining meaningful interdependencies.
- Cause-and-effect diagram: The prominent risks are visualized in a cause-and-effect diagram, categorizing them as critical enablers, mild enablers, independent risks, and critical dependents.

### 3.5 Strengths and limitations of the methodology

The research methodology demonstrated notable strengths through its multi-stakeholder approach, which captured diverse perspectives and insights while employing the grey DEMATEL framework to effectively model and visualize complex interrelationships among factors. The integration of quantitative and qualitative data enhanced the analytical depth and robustness of the findings. However, the study had several limitations that warrant consideration: the relatively modest sample size of 45 participants potentially constrained the generalisability of the results; the use of purposive-sampling methodology could have introduced selection bias in the data-collection process, and the stakeholder perspectives gathered could have been significantly influenced by specific cultural and geographical contexts, potentially limiting the universal applicability of the findings.

### 3.6 Data management and analysis

The collected data were organized and analyzed using spreadsheets and statistical tools. The results of the grey DEMATEL method provide actionable insights into how risks in one module propagate or mitigate risks in other modules, offering a systemic view of vulnerabilities in GAI systems.

The study adhered to ethical guidelines, ensured participant confidentiality, and obtained informed consent. The participants were briefed on the objectives of the study and measures were taken to protect the privacy and integrity of the collected data. This methodology provides a robust framework for identifying, categorizing, and analyzing the risks associated with GAI. By incorporating insights from diverse stakeholders and exploring the inter- relationships among risks, this study provided the foundation for developing strategies to address ethical challenges in GAI technologies. Although there are limitations, this comprehensive approach provides valuable insights into the evolving landscape of GAI risks and their implications.

## 4 Findings

This study focused on the significance of risk enablers in GAI systems – to three primary stakeholders: developers, end users, and policymakers and used the DEMATEL algorithm to thoroughly analyze them. The analysis highlighted the key enablers and dependents shaping the AI ecosystem and provided actionable insights for mitigating cascading risks. Using a causal-relationship graph, risk enablers were classified into four distinct zones: critical, mild, and independent enablers, and critical dependents. The Total Relation Matrix also highlighted both the direct and indirect influence that one risk enabler has over another.

### 4.1 AI developer’s perspective

**Figure 3:**
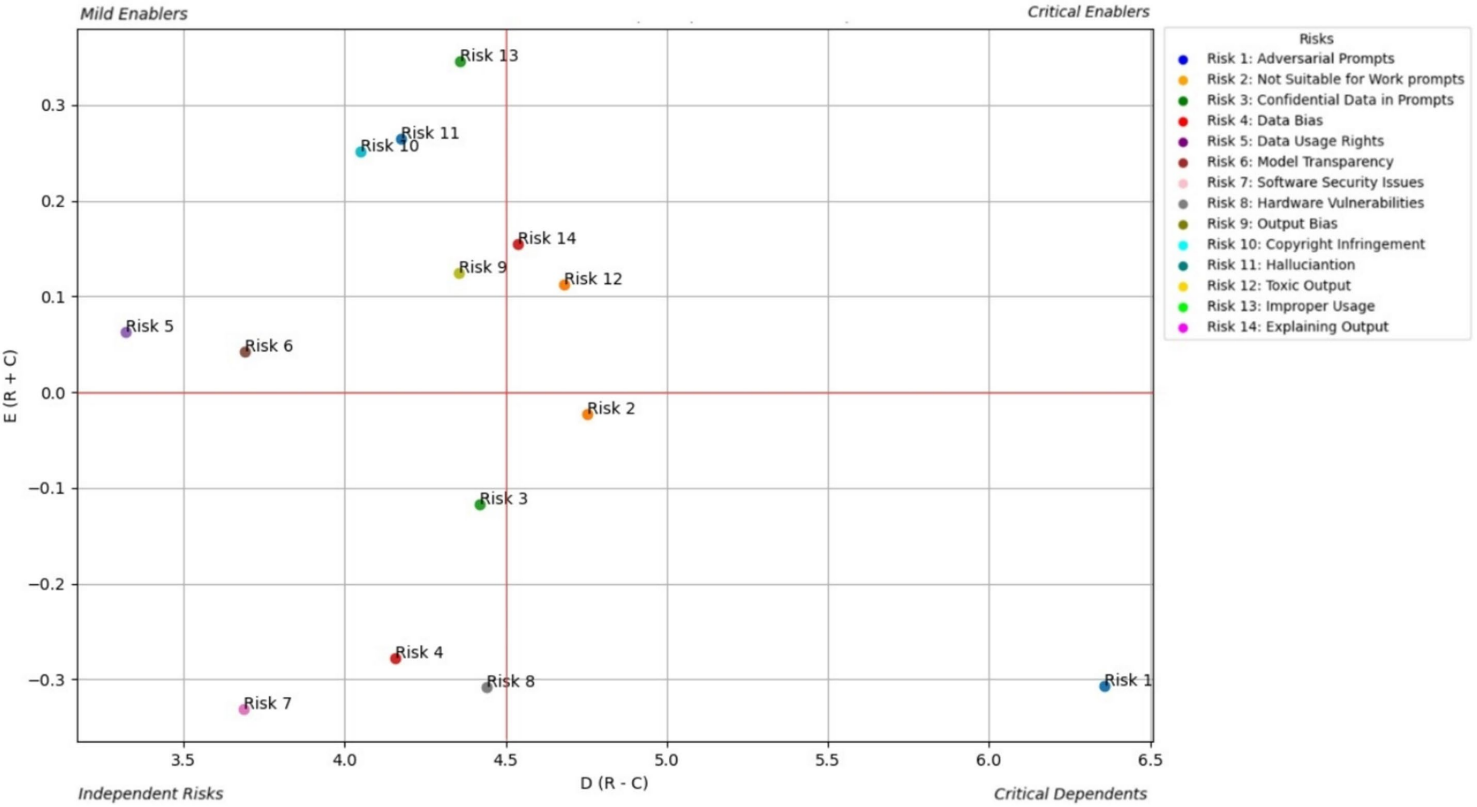
Prominent causal relationship for AI developer.

AI developers play pivotal roles in the design, development, and maintenance of GAI systems. This section presents risk enablers from a developer perspective and categorizes them into four zones based on their influence and interconnectedness. Figure 3 shows the prominent causal relationships for AI developers, and Table 2 shows the total relationship matrix for AI developer risk enablers.

**Table 2:** Total relationship matrix for AI developer risk enablers.

Denotation	RE1	RE2	RE3	RE4	RE5	RE6	RE7	RE8	RE9	RE10	RE11	RE12	RE13	RE14
RE1	0.19	0.25	0.24	0.25	0.2	0.21	0.23	0.25	0.23	0.17	0.19	0.22	0.19	0.21
RE2	0.27	0.08	0.18	0.14	0.17	0.15	0.14	0.18	0.15	0.14	0.16	0.18	0.16	0.17
RE3	0.25	0.17	0.07	0.17	0.13	0.14	0.15	0.17	0.16	0.14	0.12	0.17	0.16	0.15
RE4	0.23	0.16	0.16	0.05	0.12	0.12	0.13	0.13	0.13	0.13	0.14	0.15	0.12	0.13
RE5	0.22	0.16	0.14	0.06	0.1	0.12	0.12	0.12	0.12	0.09	0.1	0.12	0.1	0.13
RE6	0.23	0.15	0.15	0.17	0.1	0.03	0.13	0.14	0.12	0.12	0.13	0.15	0.12	0.14
RE7	0.21	0.14	0.1	0.13	0.09	0.07	0.03	0.11	0.11	0.11	0.11	0.12	0.08	0.13
RE8	0.24	0.17	0.16	0.17	0.1	0.1	0.14	0.07	0.14	0.1	0.14	0.17	0.12	0.14
RE9	0.26	0.17	0.17	0.16	0.09	0.14	0.1	0.14	0.06	0.16	0.18	0.17	0.17	0.17
RE10	0.23	0.18	0.14	0.14	0.12	0.13	0.12	0.14	0.13	0.05	0.17	0.19	0.06	0.18
RE11	0.23	0.17	0.18	0.17	0.1	0.17	0.1	0.12	0.16	0.13	0.1	0.07	0.12	0.17
RE12	0.27	0.19	0.16	0.17	0.12	0.16	0.12	0.18	0.17	0.17	0.07	0.08	0.17	0.09
RE13	0.25	0.18	0.18	0.14	0.14	0.16	0.2	0.17	0.17	0.19	0.06	0.08	0.18	0.18
RE14	0.24	0.18	0.17	0.17	0.14	0.15	0.16	0.2	0.18	0.16	0.17	0.19	0.17	0.07

From the perspective of the AI developer (Table 2; Figure 3), toxic output (RE12) is identified as the most critical and far-reaching risk in generative AI systems. DEMATEL analysis confirms this, showing that RE12 has the highest direct and total influence scores among all risks, making it a central driver within the risk ecosystem. Its impact is especially pronounced in triggering improper use (RE13) and hallucinations (RE11), with the RE12 RE13 link emerging as the strongest causal path in the study. These results suggest that unless toxic output is effectively controlled, other risks quickly escalate in severity. Developers are also particularly concerned with Explaining Output (RE14) and Adversarial Prompts (RE1), which compound the toxic output problem by either obscuring how the model arrives at harmful conclusions or actively manipulating it to generate unsafe content.

The study further highlights how these risks do not exist in isolation but interact in cascading chains. For example, RE1 (Adversarial Prompts) shows a strong causal influence on both RE12 and RE13, reinforcing how prompt manipulation can initiate toxic and improper output. Additionally, Data Bias (RE4), though categorized as a less interconnected enabler, subtly amplifies the toxic content issue by shaping the model’s response tendencies. These interdependencies signal that mitigation strategies cannot be one-dimensional. Developers must therefore prioritize multifaceted defenses—such as implementing explainable AI techniques, deploying real-time adversarial detection systems, and ensuring bias-resilient training data—to address these overlapping vulnerabilities holistically. This systemic view is essential for building safer and more reliable generative AI applications.

### 4.2 AI end user’s perspective

**Figure 4:**
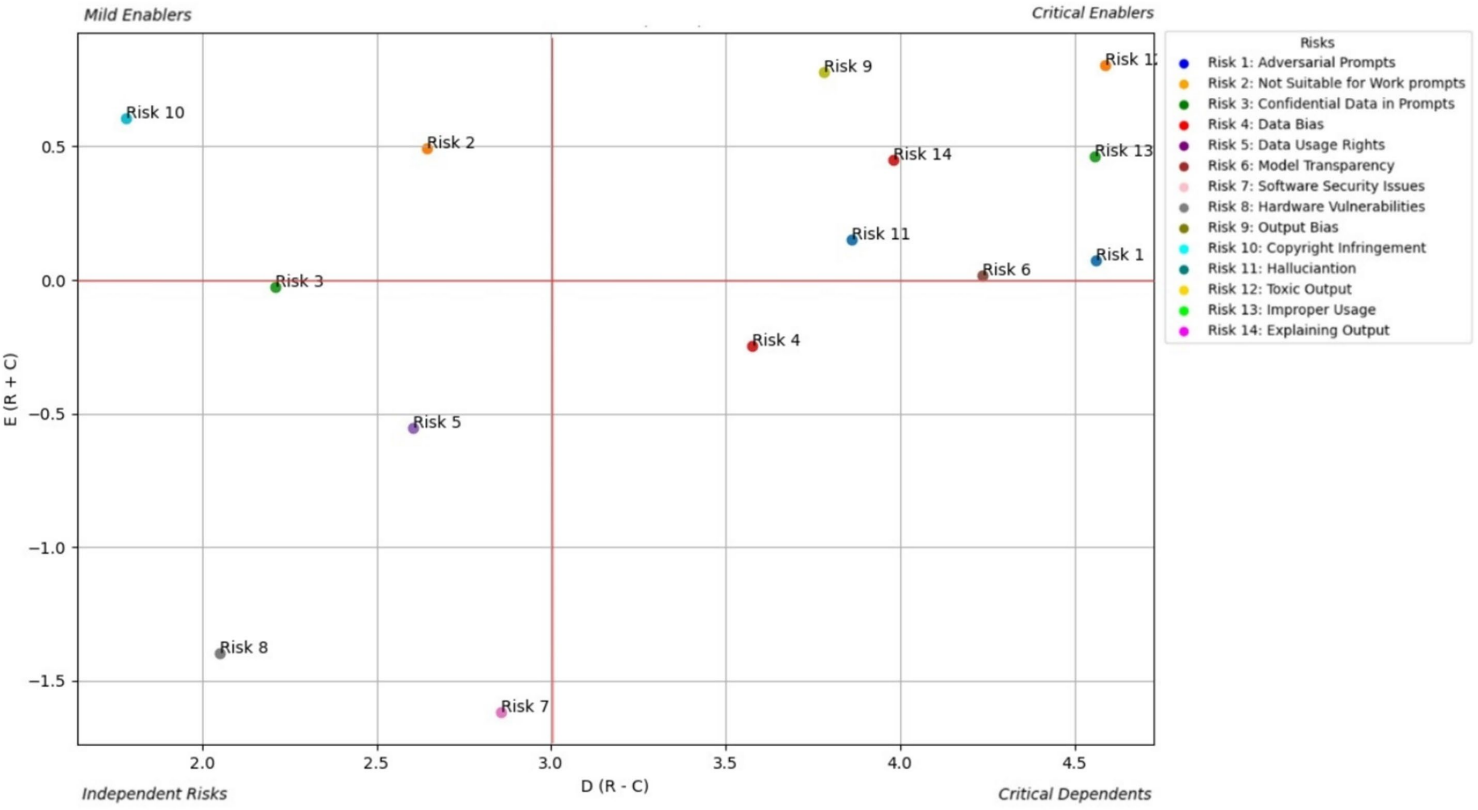
Prominent causal relationship for AI end user.

AI end users experience the downstream effects of the risks embedded within GAI systems. Figure 4 shows the prominent causal relationship for the AI end user and Table 3 shows the total relationship matrix for the AI end user risk enablers.

**Table 3:** Total relationship matrix for AI end user risk enablers.

Denotation	RE1	RE2	RE3	RE4	RE5	RE6	RE7	RE8	RE9	RE10	RE11	RE12	RE13	RE14
RE1	0.13	0.13	0.13	0.2	0.13	0.2	0.22	0.19	0.16	0.04	0.19	0.24	0.2	0.17
RE2	0.22	0.04	0.07	0.12	0.07	0.14	0.18	0.1	0.08	0.03	0.07	0.2	0.16	0.07
RE3	0.11	0.04	0.02	0.06	0.09	0.12	0.14	0.13	0.05	0.02	0.08	0.07	0.1	0.07
RE4	0.15	0.06	0.07	0.08	0.11	0.11	0.16	0.1	0.14	0.04	0.14	0.14	0.16	0.11
RE5	0.07	0.05	0.07	0.05	0.1	0.11	0.1	0.05	0.1	0.06	0.1	0.06	0.1	0.09
RE6	0.19	0.08	0.11	0.18	0.15	0.09	0.12	0.07	0.15	0.07	0.21	0.17	0.14	0.13
RE7	0.06	0.02	0.06	0.04	0.04	0.06	0.02	0.06	0.03	0.01	0.1	0.04	0.04	0.05
RE8	0.03	0.01	0.01	0.02	0.02	0.02	0.03	0.01	0.01	0.01	0.05	0.05	0.05	0.02
RE9	0.21	0.09	0.09	0.25	0.16	0.23	0.21	0.05	0.06	0.04	0.18	0.21	0.19	0.1
RE10	0.11	0.05	0.05	0.07	0.17	0.16	0.13	0.06	0.04	0.03	0.1	0.07	0.13	0.07
RE11	0.23	0.18	0.09	0.17	0.14	0.18	0.15	0.06	0.12	0.06	0.15	0.2	0.23	0.19
RE12	0.29	0.17	0.11	0.27	0.18	0.22	0.25	0.19	0.21	0.06	0.2	0.19	0.23	0.19
RE13	0.24	0.16	0.13	0.21	0.16	0.21	0.22	0.18	0.17	0.06	0.23	0.23	0.19	0.13
RE14	0.2	0.08	0.11	0.2	0.14	0.23	0.2	0.14	0.17	0.05	0.22	0.19	0.19	0.1

From the end user’s standpoint (Table 3; Figure 4), the most prominent risks are Improper Usage (RE13), Toxic Output (RE12), and Explaining Output (RE14)—all of which directly affect the trust, safety, and fairness of generative AI interactions. The study shows that these risks are not only highly visible to users but also closely interconnected, forming a dense web of concerns. RE13, which includes the potential for misinformation, fraud, or malicious use of AI-generated content, is often a result of RE12, as users may intentionally or unintentionally exploit harmful outputs. Meanwhile, the lack of clear explanations (RE14) leaves users unable to understand, question, or trust AI-generated decisions or responses, especially in high-impact contexts like financial recommendations, health advice, or educational support. These risks directly affect how users perceive and rely on AI, especially when the consequences of misinterpretation are severe.

The study emphasizes that explainability acts as a bridge between user empowerment and broader system integrity. When users cannot understand why an output was generated, they are more vulnerable to misusing or misjudging its intent, especially in emotionally or socially sensitive scenarios. This is further compounded by Data Bias (RE4) and Output Bias (RE9), which may not be immediately visible to users but subtly distort results in ways that reinforce stereotypes or exclude underrepresented groups. While users may not always articulate these risks in technical terms, their consequences manifest clearly—through frustration, disengagement, or even harm. The analysis underscores that to build trustworthy generative AI experiences, developers and regulators must focus on user-centric safeguards: clearer disclosures, content filters, feedback mechanisms, and transparent user interfaces that empower users to engage safely and meaningfully with AI outputs.

### 4.3 AI policy maker’s perspective

**Figure 5:**
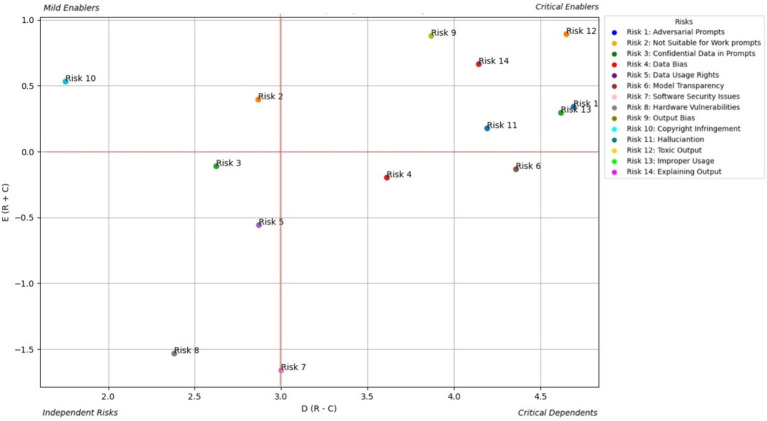
Prominent causal relationship for AI policy maker.

AI policymakers focus on the regulatory and governance aspects of GAI systems. Figure 5 shows the prominent causal relationship for AI End-policymakers and Table 4 shows the total relationship matrix for AI policymaker risk enablers.

**Table 4:** Total relationship matrix for AI policy maker risk enablers.

Denotation	RE1	RE2	RE3	RE4	RE5	RE6	RE7	RE8	RE9	RE10	RE11	RE12	RE13	RE14
RE1	0.14	0.16	0.16	0.21	0.15	0.21	0.23	0.2	0.16	0.05	0.23	0.24	0.21	0.17
RE2	0.22	0.04	0.08	0.1	0.1	0.13	0.16	0.12	0.08	0.03	0.12	0.16	0.1	0.09
RE3	0.11	0.05	0.03	0.07	0.09	0.1	0.14	0.13	0.05	0.03	0.09	0.13	0.1	0.1
RE4	0.14	0.07	0.08	0.07	0.14	0.18	0.17	0.11	0.13	0.04	0.15	0.13	0.16	0.15
RE5	0.08	0.07	0.07	0.06	0.04	0.12	0.11	0.1	0.05	0.1	0.1	0.1	0.09	0.12
RE6	0.18	0.09	0.12	0.16	0.15	0.11	0.2	0.12	0.1	0.07	0.19	0.17	0.19	0.13
RE7	0.06	0.03	0.07	0.04	0.04	0.07	0.03	0.06	0.03	0.01	0.11	0.06	0.07	0.07
RE8	0.03	0.01	0.06	0.02	0.02	0.03	0.03	0.01	0.01	0.01	0.04	0.03	0.05	0.02
RE9	0.2	0.1	0.13	0.24	0.17	0.23	0.22	0.09	0.08	0.04	0.22	0.21	0.2	0.18
RE10	0.09	0.05	0.05	0.06	0.16	0.13	0.12	0.06	0.04	0.03	0.11	0.07	0.13	0.08
RE11	0.22	0.09	0.11	0.18	0.16	0.2	0.21	0.1	0.11	0.06	0.15	0.2	0.23	0.17
RE12	0.28	0.18	0.1	0.2	0.14	0.22	0.2	0.15	0.14	0.05	0.2	0.23	0.2	0.18
RE13	0.22	0.18	0.13	0.2	0.16	0.21	0.22	0.1	0.13	0.06	0.22	0.23	0.19	0.14
RE14	0.21	0.11	0.14	0.19	0.16	0.26	0.21	0.17	0.17	0.05	0.23	0.2	0.2	0.1

From a policymaker’s perspective (Table 4; Figure 5), the most pressing risks posed by generative AI are Toxic Output (RE12), Improper Usage (RE13), and Regulatory Non-Compliance (RE10). These risks intersect at the heart of public trust, legal liability, and societal well-being. Policymakers are particularly concerned with how toxic or misleading AI-generated content can be misused at scale—for instance, to spread misinformation, automate discriminatory decisions, or circumvent legal safeguards. The study identifies a strong causal relationship from RE12 to RE13, affirming that harmful outputs increase the likelihood of non-compliant and abusive usage, which may undermine public confidence and regulatory stability. In addition, lack of transparency (RE14) exacerbates this challenge by making it difficult for institutions to audit decisions or trace accountability across AI deployments.

The study also highlights how regulatory risks are tightly woven into other systemic vulnerabilities, such as Data Bias (RE4) and Output Bias (RE9), which can lead to outcomes that violate fairness, privacy, or safety laws. For example, biased training data can produce outputs that unintentionally breach anti-discrimination regulations, while hallucinated content (RE11) can mislead consumers or violate advertising standards. These risks are particularly complex because they operate on technical, ethical and legal dimensions, requiring cross-functional responses that go beyond traditional compliance. Policymakers are therefore urged to promote cross-stakeholder alignment, invest in standards for explainability, robustness, and traceability, and develop regulatory frameworks, such as updates to the EU AI Act, that are dynamic enough to evolve with the generative AI landscape. The study reinforces that regulatory foresight, combined with adaptive enforcement mechanisms, will be essential to safeguard the public while enabling innovation.

All three stakeholder groups—developers, end users, and policymakers—agree on the significance of certain risks; however, they diverge in their emphasis and perception of the root causes. Toxic Output (RE12) and Improper Usage (RE13) consistently ranked high across all groups. Developers prioritize technical issues such as Adversarial Prompts (RE1) and Explainability (RE14), whereas end users are more affected by Output Bias (RE9) and Lack of Transparency (RE6), which impact trust and usability. Policymakers emphasize broader societal risks like Data Bias (RE4), Regulatory Non-Compliance (RE10), and Confidentiality (RE3) due to their implications for fairness and legal accountability. The comparative analysis is further deepened using Euclidean distance approach in the next section.

### 4.4 Euclidean distance analysis of stakeholder perspectives on risk enablers

**Figure 6:**
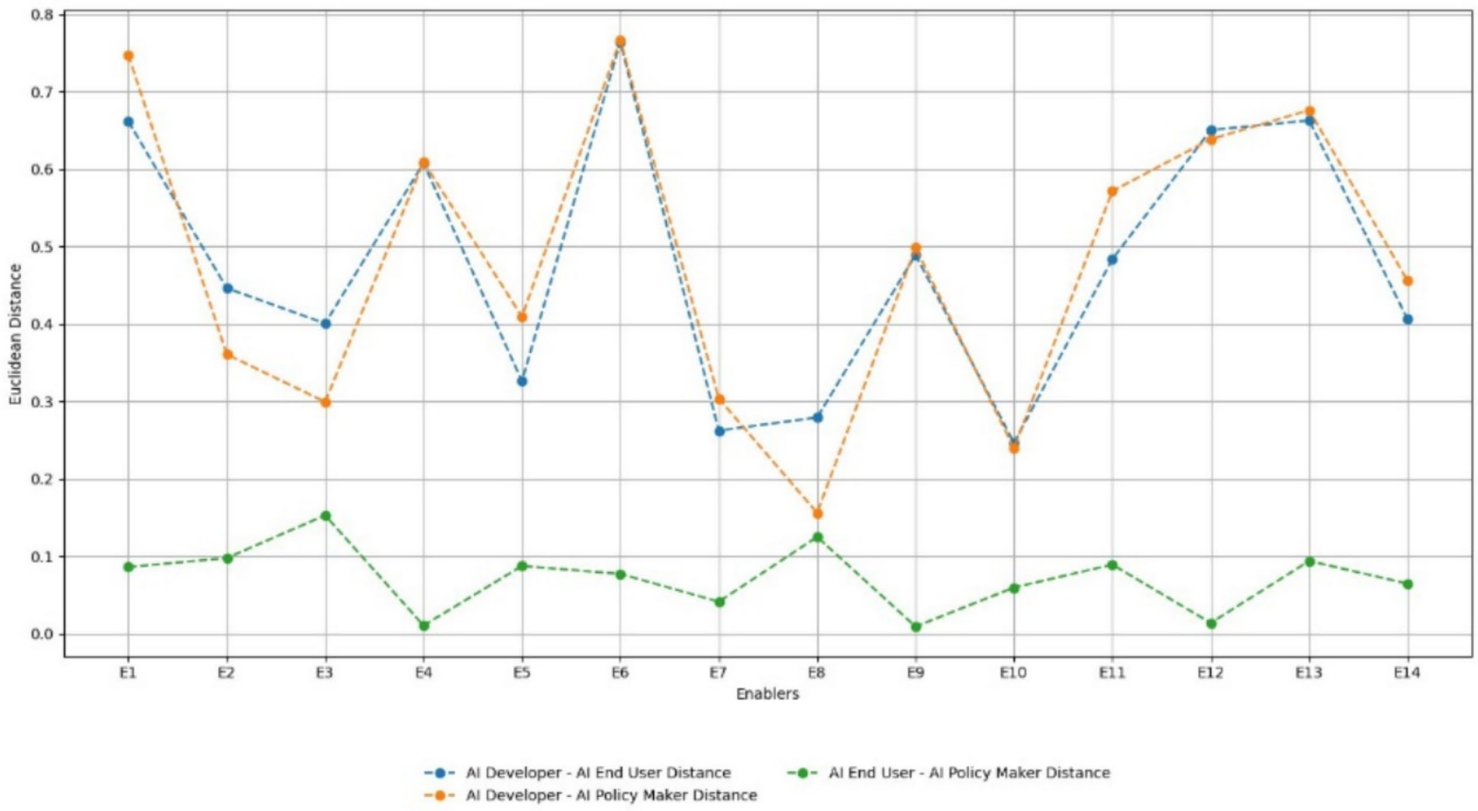
Euclidean distance between stakeholders for each risk enabler.

The Euclidean distance analysis Figure 6 offers a quantitative lens to assess how closely aligned or divergent the views of AI developers, end users, and policymakers are in relation to key generative AI risks. Adversarial Prompts (RE1), Data Bias (RE4), and Output Bias (RE9) emerged as the most divisive enablers, with high Euclidean distances indicating strong variation in perceived criticality. Developers, for instance, tend to view adversarial prompts and data bias as technical design challenges that can be mitigated through architecture improvements or fine-tuning, while policymakers and end users associate these risks with broader social harms, such as manipulation, misinformation, and discrimination. These differences suggest a disconnect between how risks are engineered and how they are experienced or regulated.

In contrast, Hardware Vulnerabilities (RE8) and Confidential Data in Prompts (RE3) showed low distance values, signaling broad consensus across all stakeholder groups. These risks are universally recognized due to their tangible and measurable impact, especially in enterprise and high-risk environments. Interestingly, Explaining Output (RE14) and Model Transparency (RE6) occupied a mid-distance zone, where stakeholders agreed on their importance but diverged on their depth of implementation or responsibility. For example, while developers may advocate for explainability features within technical constraints, users and regulators may demand deeper interpretability and accountability mechanisms.

These findings reveal that perception gaps are most prominent in areas where risks straddle technical, ethical, and regulatory boundaries. As such, the analysis underscores the need for collaborative risk framing and co-designed governance mechanisms that reflect the values and constraints of all stakeholders. Bridging these gaps is not only essential for effective regulation but also for fostering trust, inclusivity, and sustainable innovation in the generative AI ecosystem.

## 5 Discussion

The findings of this study underscore the pressing need to address multifaceted risks associated with GAI systems. While existing research has explored individual risks such as toxic outputs, hallucinations, and explainability gaps, this study highlights the interconnected and cascading nature of these risks, which exacerbates their impact across different stakeholder groups.

### 5.1 Emerging risks in generative AI

One significant gap in the current framework is the limited focus on emerging risks. For example, data poisoning, wherein malicious actors inject harmful data into training datasets, compromises the model integrity and amplifies bias. Similarly, the misuse of synthetic media, such as deepfakes, has far-reaching implications for misinformation campaigns, electoral manipulations, and reputational harm. Addressing these risks requires dynamic approaches, including continuous model monitoring, robust adversarial training, and collaborative threat intelligence sharing across stakeholders.

### 5.2 Stakeholder-specific strategies

**Figure 7:**
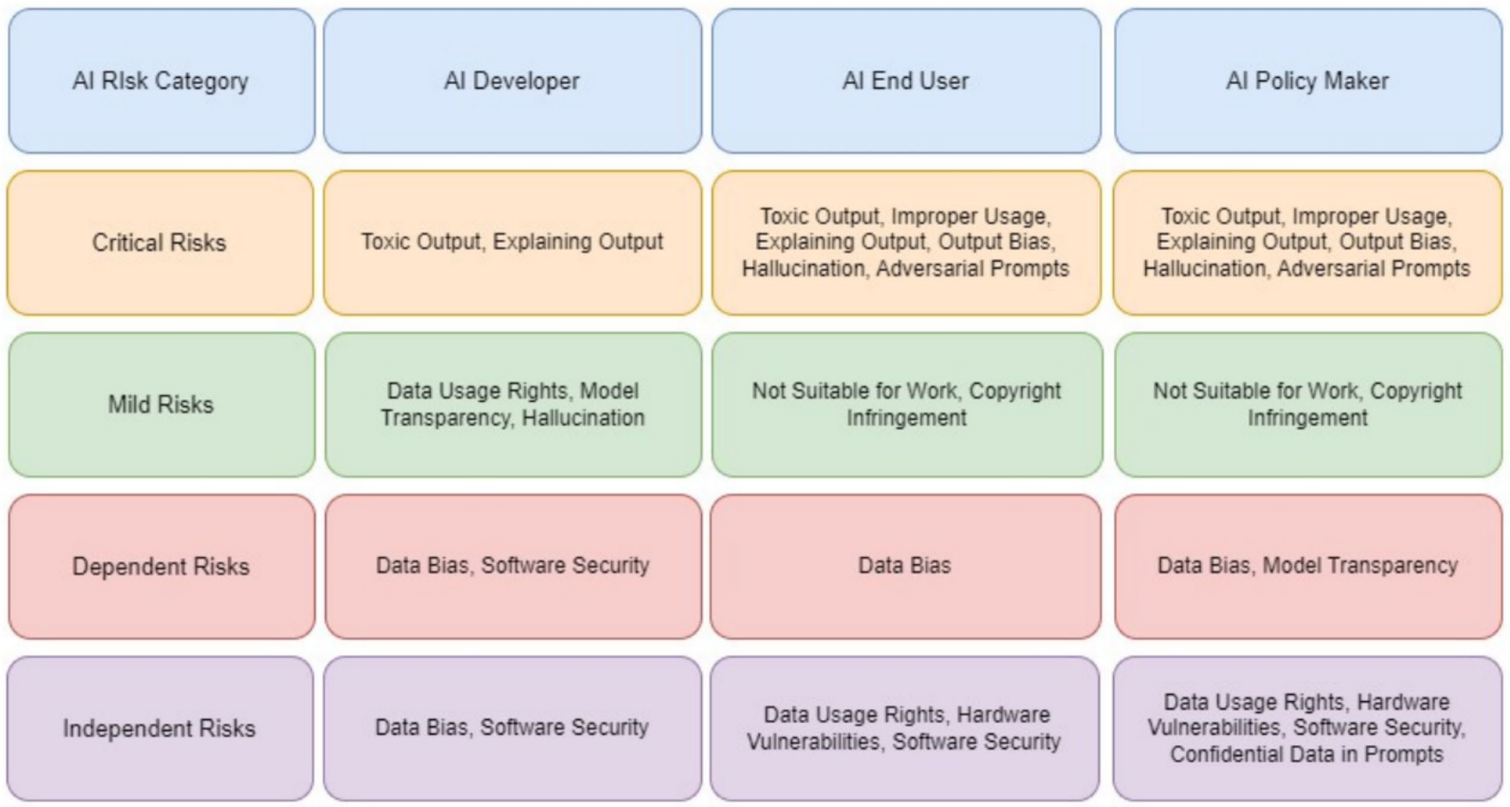
Multi-stakeholder risk assessment matrix for generative AI.

The stakeholder-based analysis of GAI risks revealed critical patterns across developers, end users, and policymakers, as shown in Figure 7. Recent incidents involving LLMs highlight how critical risks manifest in production systems, whereas cases of AI system anthropomorphisation demonstrate the challenges in explaining AI outputs. Financial institutions that implement GAI face unique challenges in balancing model transparency with security requirements. Healthcare providers who use AI for diagnosis must navigate the complex interplay between output bias and patient safety. The analysis showed that developers prioritize technical robustness, end users emphasize practical safety, and policymakers focus on societal impact. The widespread adoption of AI-powered code-generation tools illustrates the tension between copyright concerns and developer productivity. This multi-stakeholder framework suggests several promising research directions: developing quantitative metrics for Toxic Output (RE12) measurements, creating standardized explainability frameworks for LLMs, and designing cross-stakeholder risk-assessment tools. Future research should explore how these risk patterns evolve with emerging technologies such as multimodal AI systems, quantum-computing integration, and federated learning. Industries such as autonomous vehicles, drug discovery, and financial services could benefit from stakeholder-specific risk-mitigation strategies aligned with this framework. The findings underscore the need for collaborative approaches to risk management in which technical innovation meets ethical considerations and regulatory compliance.

The implications of these risks vary across stakeholder groups, necessitating tailored strategies: Developers should prioritize building robust models that can withstand adversarial attacks using techniques like adversarial testing and dynamic dataset validation. Tools, such as SHAP and LIME, can enhance explainability, enabling developers to effectively detect and mitigate biases and hallucinations (Schneider, 2024).

Policymakers must advocate real-time regulatory frameworks, including ethical audits, algorithmic transparency requirements, and standards for AI accountability. Cross-border regulatory cooperation can address the global nature of GAI risks (Raman et al., 2025).

End-users require improved literacy programs to recognize AI-generated content and its potential biases, thereby empowering them to interact responsibly with these systems. The provision of user feedback mechanisms can also enhance accountability (Ng et al., 2021).

### 5.3 Cross-domain risk manifestation

While this study emphasizes risks in critical sectors such as healthcare and finance, considering cross-domain applications is essential. For instance, in defense, GAI is increasingly used for threat simulations, where errors can have catastrophic consequences. Similarly, in education, biased-content generation in curriculum design can perpetuate systemic inequality. Understanding the domain-specific manifestations of risks ensures that mitigation strategies are contextually relevant and impactful.

### 5.4 Operationalizing risk mitigation

Operational frameworks must be developed to translate these insights into actionable solutions (Solanki et al., 2023). For example, differential privacy techniques can safeguard sensitive data in training models, whereas real-time monitoring systems can detect and flag toxic outputs. Fact-checking layers integrated into GAI systems can address hallucinations, especially in high-stakes applications such as legal and medical domains. Establishing industry benchmarks for content moderation and ethical AI practices can enhance trust and accountability.

### 5.5 Ethical and societal implications

GAI risks extend beyond technical failures and considerably affect the societal structure. Biased algorithms can reinforce existing inequalities, whereas misinformation propagated through synthetic media undermines democratic processes (Uuk et al., 2024). Policymakers must prioritize addressing these systemic issues by promoting inclusivity in training datasets and ensuring diverse representations of AI governance. Collaborative efforts among academia, industry, and civil society are vital for balancing innovation with ethical considerations.

### 5.6 Feedback loops and cascading risks

This study highlights the feedback loops among risks, such as toxic outputs, thus reinforcing data bias, which, in turn, exacerbates trust deficits among users. Breaking these cycles requires systemic interventions such as interdependent risk dashboards that monitor and mitigate cascading risks in real time. Incorporating modular AI architectures can also help isolate and address specific vulnerabilities without compromising the entire system.

This discussion highlights the dynamic and interconnected risks associated with GAI, emphasizing the need for proactive multi-stakeholder interventions. Addressing current and emerging risks requires a combination of technical innovation, regulatory oversight, and societal awareness. By operationalizing the insights from this study, stakeholders can build a more ethical, transparent, and resilient GAI ecosystem.

### 5.7 Limitations

Although this study provided a comprehensive framework for understanding the risks associated with GAI, some limitations remain. First, the participant sample, although representative of key stakeholder groups, such as developers, policymakers, and end users, was relatively small and geographically concentrated, which could limit the generalisability of our findings to broader global contexts. Expanding the sample size and including more diverse perspectives from underrepresented regions and industries could enhance the robustness of future research.

Second, the taxonomy primarily addresses generic risks and interdependencies, which may not fully capture the nuances of domain-specific challenges. For instance, sectors such as defense, education, and entertainment face unique risks not explored in detail in this study. Adapting the taxonomy to reflect these domain-specific requirements is the next essential step.

Third, the findings are based on the current state of GAI technologies and their associated risks. However, as the landscape of GAI evolves, emerging risks, such as data poisoning, synthetic-identity creation, advancements in multimodal AI, synthetic-media misuse, and advanced adversarial attacks, pose new challenges that demand proactive attention. Periodic updates to taxonomy and findings are necessary to maintain their relevance and applicability in the face of technological advancements.

Fourth, although this study identified the interdependencies among risks, it did not quantify or simulate their cascading effects. A more rigorous quantitative modeling approach, such as computational simulations, would provide deeper insights into risk propagation and amplification dynamics.

Fifth, geopolitical and regulatory variations across regions have not yet been fully addressed. GAI risks and mitigation strategies may differ significantly based on the legal, cultural, and societal contexts. Future research should explore how these differences influence the prioritization and management of AI risks.

Finally, the ethical and societal implications of GAI adoption, particularly its long-term impact on marginalized populations, public trust, and systemic inequalities, have not been thoroughly examined. Integrating multidisciplinary perspectives from fields such as sociology, philosophy, and public policy would provide a more holistic understanding of these broader implications.

Despite these limitations, this study provides a strong foundation for further exploration and actionable insights for stakeholders. Future research should address these gaps to advance the ethical, transparent, and responsible deployment of GAI systems.

## Data Availability

The raw data supporting the conclusions of this article will be made available by the authors, without undue reservation.
